# Combination antiangiogenic tyrosine kinase inhibition and anti‐PD1 immunotherapy in metastatic renal cell carcinoma: A retrospective analysis of safety, tolerance, and clinical outcomes

**DOI:** 10.1002/cam4.3812

**Published:** 2021-03-01

**Authors:** Andrew L. Laccetti, Benjamin Garmezy, Lianchun Xiao, Minas Economides, Aradhana Venkatesan, Jianjun Gao, Eric Jonasch, Paul Corn, Amado Zurita‐Saavedra, Landon C. Brown, Chester Kao, Emily N. Kinsey, Rajan T. Gupta, Michael R. Harrison, Andrew J. Armstrong, Daniel J. George, Nizar Tannir, Pavlos Msaouel, Amishi Shah, Tian Zhang, Matthew T. Campbell

**Affiliations:** ^1^ Genitourinary Oncology Service Department of Medicine Memorial Sloan Kettering Cancer Center New York NY USA; ^2^ Department of Cancer Medicine University of Texas M.D. Anderson Cancer Center Houston TX USA; ^3^ Department of Genitourinary Medical Oncology University of Texas M.D. Anderson Cancer Center Houston TX USA; ^4^ Department of Internal Medicine McGovern Medical School at UTHealth Houston TX USA; ^5^ Department of Radiology University of Texas M.D. Anderson Cancer Center Houston TX USA; ^6^ Division of Medical Oncology Department of Medicine Duke University Durham NC USA; ^7^ Duke Cancer Institute Center for Prostate and Urologic Cancers Durham NC USA; ^8^ Department of Radiology Duke University Durham NC USA

**Keywords:** combination, immunotherapy, metastatic renal cell carcinoma, salvage therapy, tyrosine kinase inhibitor

## Abstract

**Introduction:**

Two separate antiangiogenic tyrosine kinase inhibitors (TKIs) and immunotherapy (IO) combinations are FDA‐approved as front‐line treatment for metastatic renal cell carcinoma (mRCC). Little is known about off‐protocol and post‐front‐line experience with combination TKI–IO approaches.

**Methods:**

We conducted a retrospective analysis of mRCC patients who received combination TKI–IO post‐first‐line therapy between November 2015 and January 2019 at MD Anderson Cancer Center and Duke Cancer Institute. Chart review detailed patient characteristics, treatments, toxicity, and survival. Independent radiologists, blinded to clinical data, assessed best radiographic response using RECIST v1.1.

**Results:**

We identified 48 mRCC patients for inclusion: median age 65 years, 75.0% clear cell histology, 68.8% IMDC intermediate risk, and median two prior systemic therapies. TKI–IO combinations included nivolumab–cabozantinib (N +C; 24 patients), nivolumab–pazopanib (N+P; 13), nivolumab–axitinib (6), nivolumab–lenvatinib (2), and nivolumab–ipilimumab–cabozantinib (3). The median progression‐free survival was 11.6 months and the median overall survival was not reached. Response data were available in 45 patients: complete response (CR; n = 3, 6.7%), partial response (PR; 20, 44.4%), stable disease (SD; 19, 42.2%), and progressive disease (3, 6.7%). Overall response rate was 51% and disease control rate (CR+PR+SD) was 93%. Only one patient had a grade ≥3 adverse event.

**Conclusion:**

To our knowledge, this is the first case series reporting off‐label use of combination TKI–IO for mRCC. TKI–IO combinations, particularly N+P and N+C, are well tolerated and efficacious. Although further prospective research is essential, slow disease progression on IO or TKI monotherapy may be safely controlled with addition of either TKI or IO.

## Graphical abstract

Real world use of combination anti‐angiogenic tyrosine kinase inhibitor (TKI)—anti‐PD1 immunotherapy (IO) is feasible, well tolerated and efficacious for metastatic renal cell carcinoma in the second line setting and beyond. Although further prospective research is essential to define optimal use of salvage TKI‐IO, slow disease progression on IO or TKI monotherapy may be safely controlled with addition of either a TKI or IO.

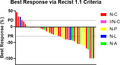

## 1 INTRODUCTION

Antiangiogenic tyrosine kinase inhibitors (TKIs) and immunotherapies (IO) targeting programmed cell death protein 1 (PD‐1) have revolutionized the treatment of metastatic renal cell carcinoma (mRCC). Two TKI–IO combinations have been approved by the Food and Drug Administration (FDA) to date for front‐line treatment of advanced RCC: avelumab combined with axitinib^1^ and pembrolizumab combined with axitinib.^2^ Both combination therapies exhibit increased progression‐free survival (PFS) compared to sunitinib; pembrolizumab plus axitinib also demonstrates improvement in overall survival (OS). An early press release for the phase III CheckMate‐9ER trial has reported that combination nivolumab and cabozantinib compared to sunitinib in patients with previously untreated advanced or metastatic RCC has met its primary endpoint of PFS at final analysis, in addition to secondary endpoints of OS and objective response rate (ORR) at a prespecified interim analysis.^3^

TKI–IO combinations are not the only option for front‐line management of mRCC. Many patients are treated with either combination ipilimumab–nivolumab, IO monotherapy, or TKI monotherapy. Most of these patients develop eventual progression and a need for effective subsequent treatments establishing a potential role for TKI–IO in the second line setting and beyond. Although clinical trials provide an idealized environment to investigate treatment efficacy and safety, they have limited capacity to generalize findings to larger, more inclusive populations of patients, providers, and health care delivery systems.^4^ Critical evaluation of real‐world evidence is essential to accurately characterize TKI–IO prescribing patterns, treatment tolerance, and efficacy. In order to better inform patient selection, dosing, expected toxicity, and future prospective trial design, we present herein the first retrospective analysis of real‐world, off‐label TKI–IO use for mRCC with a focus on prescribing patterns, tolerance, and clinical outcomes.

## 2 MATERIALS AND METHODS

We conducted a retrospective analysis of consecutive patients with mRCC treated with off‐label combination TKIs and a PD‐1 inhibitor‐based immunotherapy (IO) between November 2015 and January 2020 at the University of Texas MD Anderson Cancer Center (MDACC) and Duke Cancer Institute. All patients were treated in the second‐line or beyond setting. Patients were identified for inclusion by medical oncologists and advanced practice providers managing RCC patients at the two institutions. All patients who had at least one additional visit to confirm initiation of TKI–IO combination were included for analysis. Three patients at MDACC who received pembrolizumab in combination with axitinib as subsequent‐line treatment were identified but excluded from the analysis. In line with standard IO and TKI monotherapy practice, patients with a history of clinically significant autoimmune disease or a cardiovascular event (i.e. myocardial infarction) within 6 months were not administered TKI–IO combination treatments, and, therefore, were not included in this study.

Basic demographic elements and information related to diagnosis and treatment were collected through review of the patients' electronic medical record (EMR): age, gender, race, co‐morbidities, histology, stage, International Metastatic RCC Database Consortium (IMDC) risk scores, treatment history, performance status, outcomes, and survival data. Data specific to treatment with TKI–IO were collected including drug combination, dose, line of systemic therapy, time from initial diagnosis to initiation of TKI–IO, duration of prior TKI or IO monotherapy (before start of combination), associated adverse events (with CTCAE version 5.0 grading estimated from chart documentation), and duration of combination therapy. Clinical progression was also designated when documented in the EMR. A blinded board‐certified radiologist reviewed all restaging images to assess best radiographic response as defined by RECIST v1.1^5^ and, when applicable, date of progression. The primary objective of this study was to report the response rate for post‐front‐line TKI–IO combinations. Secondary objectives included determining PFS, OS, and toxicity of TKI–IO therapies. Approval from the MDACC and Duke Institutional Review Boards was obtained prior to study initiation and all clinical data was collected and stored according to best practices, protecting patient confidentiality and data integrity.

### 2.1 Statistical analysis

Categorical variables were tabulated with frequency and percentage, and continuous variables were summarized using descriptive statistics. GraphPad Prism Version 8.0.0 was used to calculate the following statistics. An estimated median follow‐up was calculated by the reverse Kaplan–Meier method. Survival analysis was performed by calculating time from TKI–IO initiation to last follow‐up or death. The Kaplan–Meier method and log rank test were applied for survival analysis.

## 3 RESULTS

Fifty‐two patients were identified for study inclusion. Four patients were excluded on the basis of incomplete follow‐up data or inability to confirm receipt of combination TKI–IO. In total, 48 patients were analyzed: median age at initiation of combination therapy 65.0 years (range 36–86), 85.4% Caucasian, and 79.2% male. 75.0% of cases were clear cell histology, 79.2% included prior nephrectomy, and 79.2% were IMDC intermediate or poor risk disease. The majority of patients were heavily pretreated with 77.1% having received at least 2 lines of systemic therapy prior to initiation of combination TKI–IO; in patients who had received single agent IO or TKI that was later part of combination therapy, this was included as a prior line of therapy (see Table 2). At initiation of combination therapy, 77.1% of patients had at least two metastatic sites of disease. For complete baseline patient characteristics please see Table 1.

**TABLE 1:** Patient and disease characteristics (N = 48)

Patient characteristics	No.	Percent of cohort (%)
Age (Years)		
≥65	25	52.1
<65	23	47.9
Gender		
Male	38	79.2
Female	10	20.8
Race		
Caucasian	41	85.4
Hispanic	2	4.2
Black	3	6.3
Asian/Pacific Islander	2	4.2
ECOG performance status		
0	27	56.3
1	16	33.3
2	5	10.4
Stage at Diagnosis		
Non‐metastatic	18	37.5
Metastatic	30	62.5
Histology		
Clear Cell	36	75.0
Papillary	9	18.8
Chromophobe	1	2.1
Sarcomatoid	1	2.1
Sarcomatoid/Clear Cell	1	2.1
IMDC risk (Time of TKI‐IO initiation)		
Good	10	20.8
Intermediate	33	68.8
Poor	5	10.4
Number of prior systemic therapies		
Prior Nephrectomy	38	79.2
IO Monotherapy	23	47.9
Pazopanib	20	41.7
Sunitinib	25	52.1
Cabozantinib	15	31.3
Axitinib	13	27.1
Lenvatinib	3	6.3
Bevacizumab	3	6.3
IL−2	6	12.5
mTOR Inhibitor	4	8.3
Radiation	22	45.8
Metastectomy	12	25
Embolization	4	8.3
Median no. prior treatments	2 (IQR = 2–3)	
Metastatic sites		
Lymph Node	25	52.1
Bone	21	43.8
Lung/Pleura	28	58.3
Brain	5	10.4
Adrenal Gland	8	16.7
Liver	6	12.5
Renal	3	6.3
Skin/Soft tissue	7	14.6
Peritoneum	11	22.9
Median no. metastatic sites	2 (IQR =2–3)	

ECOG, Eastern Cooperative Group; IMDC, International Metastatic RCC Database Consortium; IQR, Interquartile range.

Table 2 outlines the prescribing patterns for TKI–IO combinations. The majority of patients, 77.1% (37/48), received either nivolumab + cabozantinib (N+C, n = 24) or nivolumab + pazopanib (N+P, n = 13). The median duration on therapy was 4.8 months (IQR 3.0–8.7 months). Most patients were either on TKI or IO monotherapy prior to initiation of combination therapy.

**TABLE 2:** Characteristics of combination TKI‐IO use.

IO‐TKI Combination	No. (%)	Treatment Received First No. (%)		Median Duration of Combination IO‐TKI Therapy (Months)	Best Response No. (%)			
					CR	PR	SD	PD
Nivolumab‐Pazopanib	13 (27.1)	Pazopanib	9 (69.2)	5.8 (IQR 4.7–8.3)	0 (0)	6 (46.1)	7 (53.8)	0 (0)
		Nivolumab	4 (44.4)					
Nivolumab‐Cabozantinib	24 (50.0)	Cabozantinib	11 (45.8)	4.4 (IQR 2.4–6.3)	2 (8.3)	10 (41.7)	10 (41.7)	2 (8.3)
		Nivolumab	12 (50.0)					
		Synchronous	1 (4.2)					
Nivolumab‐ Axitinib	6 (12.5)	Axitinib	2 (33.3)	9.4 (IQR 5.0–11.3)	1 (16.7)	3 (50.0)	1 (16.7)	0 (0)
		Nivolumab	2 (33.3)					
		Synchronous	2 (33.3)					
Nivolumab‐Levantinib	2 (4.2)	Lenvatinib	2 (100.0)	5.6 (IQR 5.1–6.1)				
		Nivolumab	0 (0.0)					
Ipi/Nivo‐Cabozantanib	3 (6.3)	Ipilimumab/Nivolumab	2 (67.7)	4.0 (IQR 2.7–9.6)				
		Cabozantinib	0 (0.0)					
Total	48	IO	20 (41.7)	4.8 (IQR 3.0–8.7)				
		TKI	*25 (52.1)*					
		Synchronous	*3 (6.3)*					

IQR, Interquartile range; CR, Complete response; PR, Partial response; SD, Stable disease; PD, Progressive disease; Ipi, Ipilimumab; Nivo, nivolumab

Focusing on the two largest patient cohorts, patients treated with N+C compared to N+P had higher median metastatic sites (3 vs. 2) and were more heavily pretreated with agents unique to their TKI–IO combination (median 2 vs. 0). In the N+P group, more patients started TKI prior to addition of nivolumab at progression (69.2% vs. 45.8%). At time of combination therapy initiation, 83.3% of patients received cabozantinib 20–40 mg and 92.3% received pazopanib 200 mg. 35.1% of patients treated with these regimens required a TKI dose reduction or discontinuation during the study period.

### 3.1 Response analysis

**FIGURE 1:**
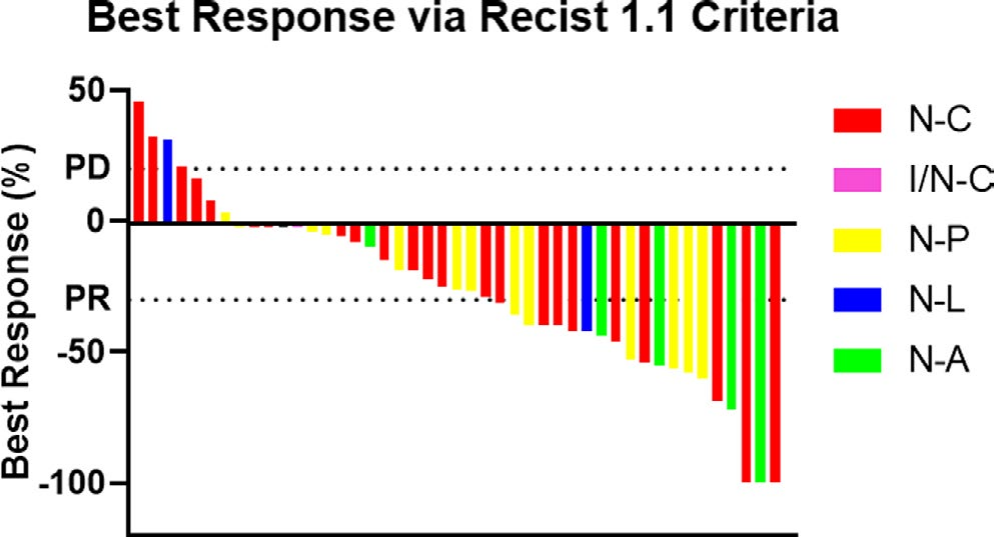
TKI–IO waterfall plot for best response as defined by RECIST 1.1. N‐C, nivolumab–cabozantinib; I/N‐C, ipilimumab/nivolumab–cabozantinib; N‐P, nivolumab–pazopanib; N‐L, nivolumab–lenvatinib; N‐A, nivolumab–axitinib.

Response data, evaluated by an independent radiology review, were available in 45 of 48 patients. Among the 45 patients, best response was as follows: complete response (CR; n = 3, 7%), partial response (PR; n = 20, 44%), stable disease (SD; n = 19, 42%) and progressive disease (PD; n = 3, 7%) (Table 2). The overall response rate (CR+PR) was 51% (95% CI: 37%, 66%) and the disease control rate (CR+PR+SD) was 93% (95% CI: 85%, 100%). Figure 1 depicts the associated waterfall plot of best objective response rates by treatment regimen.

### 3.2 Survival analysis

**FIGURE 2:**
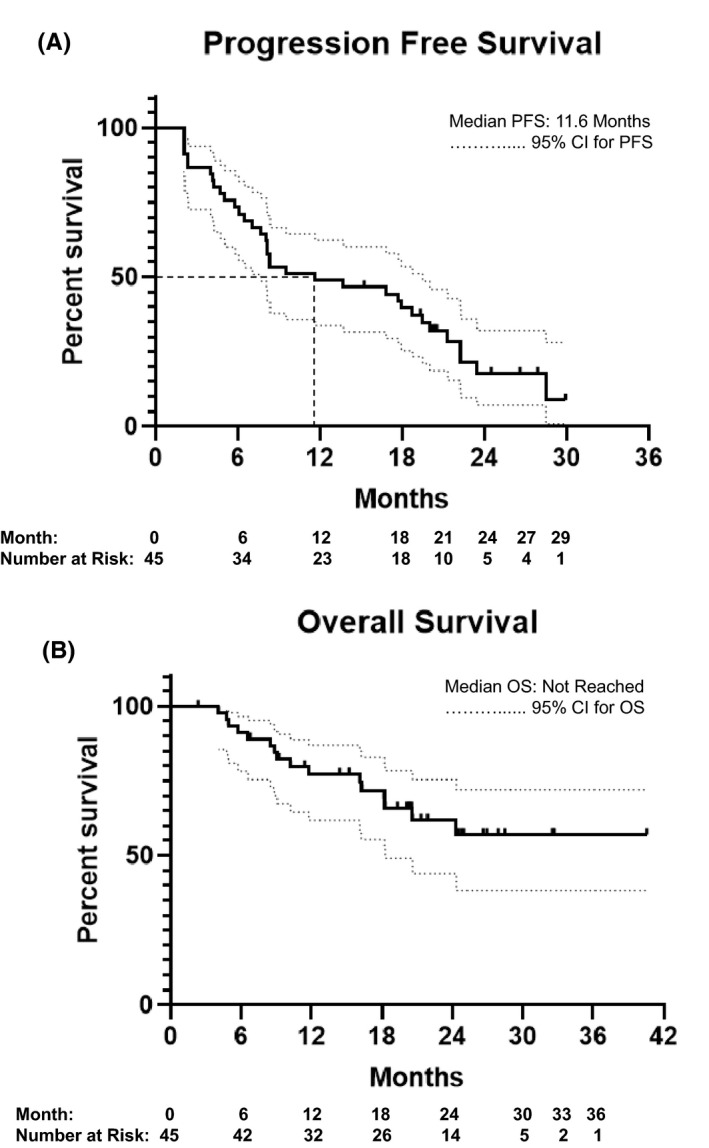
Progression‐free (A) and overall (B) survival for all TKI–IO patients. PFS, progression‐free survival; OS, overall survival; CI, confidence interval.

The estimated median follow‐up time for all patients was 21.3 months. Of the 48 patients, 45 patients had progressive disease and/or died and were included in analysis of PFS. The estimated median PFS was 11.6 months. The estimated 1‐year PFS probability was 48.9%. At the time of data cut off, 16 of the 48 patients had died and median OS was not reached. Figure 2 shows Kaplan‐Meir curves for PFS and OS.

As they were the two largest cohorts, outcomes for patients treated with N+C and N+P were analyzed in greater detail. With an estimated median follow‐up of 20.3 months, the N+C cohort had a median PFS of 7.3 months (initiated TKI first: 4.8, IO first: 8.2) and a median OS of 18.2 months (TKI first: 11.8, IO first: 24.3). The N+P cohort had an estimated median follow‐up of 21.3 months and demonstrated a median PFS of 21.3 months (TKI first: 16.5, IO first: 21.8); median OS was not reached.

### 3.3 Toxicity analysis

Grade ≥1 and ≥2 toxicities were reported in 95.8% and 21.9% of patients respectively. One patient treated with nivolumab and axitinib experienced Grade 4 hypertension. The most common adverse events were fatigue (85.4%), diarrhea (39.6%), nausea (37.5%), weight loss (31.3%), and hypertension (29.2%) (Table 3). IO was temporarily held or discontinued in 5 patients for treatment related complications including suspected IO‐associated pneumonitis (3 patients) and nephritis (1 patient). No patients permanently discontinued TKI due to side effects and 35.4% of patients required a TKI dose reduction. Of note, only one patient on N+P (pazopanib starting dose 800 mg) developed elevated transaminases.

**TABLE 3:** Adverse events reported on TKI‐IO

Adverse event	Any grade		Grade 3 (No.)	Grade 4 (No.)
	No.	%		
Fatigue	41	85.4	0	0
Diarrhea	19	39.6	0	0
Nausea	18	37.5	0	0
Weight Loss	15	31.3	0	0
Hypertension	14	29.2	0	1
Cytopenia	12	25	0	0
Mucositis	12	25	0	0
Rash	8	16.7	0	0
Hypothyroidism	7	14.6	0	0
Hand‐foot syndrome	7	14.6	0	0
Colitis	6	12.5	0	0
Pneumonitis	6	12.5	0	0
Electrolyte abnormality	5	10.4	0	0
Arthralgia	3	6.3	0	0
Proteinuria	2	4.2	0	0

## 4 DISCUSSION

To our knowledge, this is the first case series examining off‐label use of combination TKI–IO in mRCC. The MD Anderson and Duke experiences substantiate adequate feasibility, safety, and tolerance for this combination approach, in line with previously published preclinical and clinical data.^6^, ^7^, ^8^, ^9^, ^10^ The majority of patients in our cohort were heavily pretreated, IMDC intermediate risk,^11^ and exposed to either combination N+P or N+C (77.1%). Disease control was observed in 93% of patients, with a median PFS of 11.6 months. Objective responses were observed in approximately 53% of patients and were generally durable. TKI–IO was well tolerated with only 35.4% of patients requiring TKI dose reduction and 6.3% of patients necessitating discontinuation of immunotherapy, mostly for presumed pneumonitis (all of whom exhibited partial response).

The primary antitumor activity of TKIs is generally ascribed to their inhibitory effect on tumor angiogenesis; nevertheless, emerging data support an immune‐modulatory role for these agents. Increasing local levels of VEGF‐A attenuates adhesion molecule expression on endothelial cells, effectively blocking infiltration of immune cells into the tumor microenvironment.^12^ VEGF‐A also suppresses dendritic cell activity and modulates proliferation of regulatory T‐cells which inhibits CD8+ T‐cell response.^13^, ^14^, ^15^ Mouse models suggest that normalization of tumor vasculature produces increased T‐cell recruitment in tumors.^16^, ^17^ In consideration of these preclinical data, sound biologic rationale exists for mechanistic additive potential or synergy between VEGF targeted TKIs and IO agents in RCC. Further substantiating this claim, histologic analysis of mRCC samples exposed to antiangiogenic‐TKIs demonstrate increased PD‐L1 expression on tumor cells and PD‐1 expression on tumor‐infiltrating lymphocytes.^18^, ^19^, ^20^ Unfortunately, confidently differentiating a synergistic or additive effect of TKI–IO is outside the scope of this retrospective case series. Furthermore, front‐line clinical trials for axitinib–avelumab and axitinib–pembrolizumab do not robustly provide insight to this question with only 21.2% and 27.8% of patients receiving sunitinib going on to receive subsequent immunotherapy.^1^, ^2^

**FIGURE 3:**
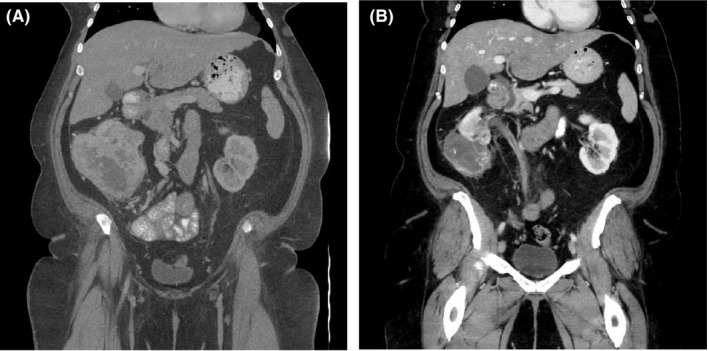
An exceptional responder pre‐(A) and post (B)‐initiation of nivolumab–pazopanib. This patient went on to have a consolidative cytoreductive nephrectomy with only 3% viable tumor returning on pathology.

JAVELIN Renal 101^1^ and KEYNOTE‐426^2^ have dramatically changed standard of care front‐line therapy for mRCC. Nivolumab–cabozantinib is also promising candidate for FDA approval (CheckMate‐9ER)^21^ as is ipilimumab–nivolumab–cabozantinib, which is currently under phase III investigation in untreated advanced and metastatic RCC (COSMIC‐313 – NCT03937219). Unlike JAVELIN Renal 101 and KEYNOTE‐426, our analysis focused on TKI–IO use in the second‐line setting and beyond. Despite 77.1% of patients in receiving at least two lines of prior systemic therapy, our observed ORR of 51% was similar to that of prospective first‐line TKI–IO trials^1^, ^2^ as well as previously published phase 1 data for nivolumab–pazopanib and nivolumab–cabozantinib in the salvage setting—45% and 36% respectively.^8^, ^22^ The METEOR trial and Checkmate 025, investigating cabozantinib and nivolumab after TKI failure, respectively, both reported an ORR of 21%,^23^, ^24^ while a pooled analysis of patients treated with second‐line TKI monotherapy after IO at MDACC and Memorial Sloan Kettering Cancer Center revealed an ORR of 41.2%.^25^ In our heavily pretreated mRCC cohort, combination TKI–IO achieved comparable, if not superior, response rates in reference to these monotherapy strategies. As further support for salvage TKI–IO use, one exceptional responder underwent consolidative cytoreductive nephrectomy following second‐line TKI–IO exposure. Pathology confirmed a near complete response with only 3% viable tumor remaining (see Figure 3).

To provide further assessment of the potential for TKI–IO to overcome resistance to TKI or IO monotherapy, we examined patients on either TKI or IO monotherapy for ≥6 months prior to TKI–IO initiation. This population of patients demonstrated long‐term stable disease or therapeutic response to TKI or IO monotherapy followed by development of treatment resistance. 22.9% of the cohort was on IO monotherapy for ≥6 months prior to initiation of TKI and demonstrated a subsequent TKI–IO response rate of 63.6% (100% disease control rate) with an associated median PFS of 18.8 months. 22.9% of patients were on TKI monotherapy for ≥6 months prior to initiation of IO and demonstrated a TKI–IO response rate of 27.3% (81.8% disease control rate) with an associated median PFS 17.9 months. Taken together, this analysis suggests that TKI–IO combination therapy has the potential to overcome TKI or IO monotherapy resistance in the second‐line setting and beyond. This effect may be more robust for IO monotherapy followed by addition of TKI considering the larger observed response rate. However, due the retrospective nature of this study and small sample size, further study of this claim is warranted.

Dissimilar to early phase TKI–IO studies utilizing cabozantinib or pazopanib, we observed significantly less toxicity in our cohort specifically with respect to transaminase elevation (detected in only one patient who was initiated on pazopanib 800 mg oral daily). Amin et al. and McDermott et al. explored pazopanib 600–800 mg daily in combination with nivolumab 2 mg/kg every 2–3 weeks. Grade 3 transaminase elevation was reported in up to 90% of patients.^8^, ^26^ The likely explanation for our discordant results lies in TKI dose selection. The majority of patients in our cohort were administered low‐dose pazopanib (200 or 400 mg daily), likely avoiding the hepatoxicity witnessed in prior studies. Previously reported data in 47 patients treated with cabozantinib 40 mg daily and nivolumab 3 mg/kg every 2 weeks found a 60% rate of transaminase elevation with 14% grade 3 or above.^8^ As most cabozantinib patients in our study received 40 mg daily, the reason for this incongruent result is less clear and possibly stems from patient selection, hepatic drug tolerance related to prior TKI use or specific sequencing of agents.^27^ Furthermore, the addition of cabozantinib to IO (or vice versa), with subsequent titration of TKI dose, may have prevented a degree of the toxicity observed with both agents started concurrently. We contend that a key feature of safe and effective combination TKI–IO use is conservative TKI dosing with up‐titration as permitted by tolerance. Treatment layering is also feasible; although, there is limited data to support this approach, a case report by Yu‐Li et al. supports the potential for pazopanib to reverse nivolumab resistance when added in parallel.^28^

We recognize several limitations inherent to this study. First, the assembled cohort was biased toward male sex, Caucasians, and clear cell histology, limiting generalizability outside these demographic and disease criteria. Second, due to limited patient numbers, we were not able to decisively comment on the differential effect of varying TKI–IO combinations. Prospective trials and expanded, multicenter analyses of real‐world data are essential to comprehensively investigate varying combination TKI–IO regimens in the salvage setting. Third, the retrospective nature of this study limited our ability to monitor medication compliance and to precisely determine grade and relatedness of toxicity. To alleviate the former issue, all patients without adequate EMR documentation of approximate timing and duration of therapy were disqualified from study inclusion. For the latter issue, subjective toxicities were graded by detailed review of symptom reporting with a focus on associated functional limitations and the need for treatment discontinuation. Of note, we utilized independent, board certified, blinded radiologists for determination of response and progression. In addition to chart review, obituary records were reviewed to accurately determine dates of death, attempting to limit any bias associated with PFS or OS. Of note, our cohort potentially reflects a narrowing population of patients within mRCC given increasing utilization of front‐line pembrolizumab–axitinib and avelumab–axitinib; nevertheless, effective sequencing approaches will be important to understand resistance to these combinations in the future. Lastly, selection bias is a potential concern, as these patients were not identified via a systematic algorithm. Even so, we feel we were able to effectively capture all patients meeting criteria for inclusion.

## 5 CONCLUSIONS

This report endorses the feasibility, safety, and efficacy of combination TKI–IO for mRCC in the second‐line setting and beyond. Complementary to level 1 evidence for front‐line axitinib combined with avelumab^1^ or pembrolizumab,^2^ our data support safety and tolerance for alternative combinations of PD‐1 inhibitors and TKIs, including nivolumab with pazopanib, cabozantinib, axitinib, or lenvatinib. mRCC patients who are heavily pretreated can tolerate combination TKI–IO when TKI dosing is conservative. Salvage TKI–IO approaches demonstrate encouraging response rates and survival, especially when compared to prospective second‐line monotherapy data.^23^, ^26^ Phase III clinical trials are currently underway to rigorously investigate optimal patient selection and dosing for cabozantinib/nivolumab (NCT03141177, NCT03937219, NCT03793166, and NCT03635892) and lenvatinib/pembrolizumab^29^ (NCT02811861). Our work also validates a rationale for prospective combination studies involving low‐dose pazopanib. While we await results of prospective trials, strong consideration should be given to off‐label TKI–IO combinations for select patients in the salvage setting even when heavily pretreated.

## CONFLICT OF INTEREST

AL has received honoraria income from Bayer. BG has no conflict of interest disclosure to report. ME has no conflict of interest disclosure to report. AV has no conflict of interest disclosure to report. JG has received consulting/advisory income from CRISPR Therapeutics AG, Pfizer, Janssen, Symphogen, AstraZeneca, Jounce Therapeutics, ARMO Biosciences, Nektar Therapeutics, and Guidepoint Global. EJ receives consulting income and research funding from Exelixis, Merck, Pfizer and consulting income from Eisai, Novartis, and Roche. PC has no conflict of interest disclosure to report. AZ‐S I has received honoraria from Bayer and AstraZeneca, and an education stipend from Pfizer in the last year. LCB has no conflict of interest disclosure to report. CK has no conflict of interest disclosure to report. ENK has no conflict of interest disclosure to report. RTG has no conflict of interest disclosure to report. MRH has received research funding from Acerta, Astellas, Astra Zeneca, Bayer, BMS, Clovis Oncology, Exelixis, Merck, Pfizer, Seattle Genetics; consulting from AstraZeneca, Bayer, BMS, Exelixis, FujiFilm, Genentech, Janssen, Pfizer; promotional speaking from Genentech. AJA receives consulting income from research funding to Duke from Pfizer/Astellas, Janssen, Genentech/Roche, Merck, Astra Zeneca, Bayer, Dendreon, and BMS. DJG has received research funding from Acerta Pharmaceuticals, Astellas, Bayer, BMS, Calithera, Exelixis, Janssen Pharmaceuticals, Novartis, Pfizer, Sanofi Aventis; Consultant or Advisory board for Astellas, AstraZeneca, Axess Oncology, Bayer H/C Pharmaceuticals, BMS, Capio Biosciences, Exelixis, Flatiron, Janssen, Merck, MJH Associates, Modra Pharmaceuticals, Myovant Sciences, Physician Education Resource LLC, Pfizer, Sanofi Aventis, and Vizuri Health Sciences LLC; Honoraria or travel fees from Bayer, EMD Serono, Exelixis, Ipsen, MJH Associates, Pfizer, Sanofi Aventis, UroGPO, and UroToday. NT receives consulting income and/or research funding from Bristol‐Myers‐Squibb, Pfizer, Nektar Therapeutics, Exelisis Inc, Eisai Medical Research, Eli Lilly, Oncorena, Ono Pharmaceutical, Calithera Bioscience, Surface Oncology, Novartis, Ipsen PM, Mirati Therapeutics, and Wpizyme Inc. PM has received honoraria for service on Scientific Advisory Board for Mirati Therapeutics, Exelixis, and BMS, consulting for Axiom Healthcare Strategies, non‐branded educational programs supported by Exelixis and Pfizer, and research funding for clinical trials from Takeda, BMS, Mirati Therapeutics, Gateway for Cancer Research, and UT MD Anderson Cancer Center. PM is also supported by a Young Investigator Award by the Kidney Cancer Association, a Career Development Award by the American Society of Clinical Oncology, by a Concept Award by the United States Department of Defense, and by the MD Anderson Khalifa Scholar Award. AS receives research support from Eisai, EMD Serono, and BMS. Honoraria income has been received by Exelixis, Pfizer and Roche/Oncology Information Group. TZ has received research funding (to Duke) from Pfizer, Janssen, Acerta, Abbvie, Novartis, Merrimack, OmniSeq, PGDx, Merck, Mirati, and Astellas; consulting/speaking with Genentech Roche, Exelixis, Genomic Health, and Sanofi Aventis; and consulting with AstraZeneca, Bayer, Pfizer, Foundation Medicine, Janssen, Amgen, BMS, MJH Associates, and IQVIA. She has stock/ownership/employment/consulting (spouse) from Capio Biosciences, Archimmune Therapeutics, and Nanorobotics. MTC has received honoraria and consulting fees from Eisai, Exelixis, EMD Serono, Pfizer, Seattle Genetics, Astellas, and AstraZeneca. Research support is provided by Exelixis, EMD Serono, Pfizer, and Janssen.

## Data Availability

The data that support the findings of this study are available from the corresponding author upon reasonable request.

## References

[1] Motzer RJ , Penkov K , Haanen J , et al. Avelumab plus axitinib versus sunitinib for advanced renal‐cell carcinoma. N Engl J Med. 2019;380:1103‐1115. 30779531 10.1056/NEJMoa1816047 PMC6716603

[2] Rini BI , Plimack ER , Stus V , et al. Pembrolizumab plus axitinib versus sunitinib for advanced renal‐cell carcinoma. N Engl J Med. 2019;380:1116‐1127. 30779529 10.1056/NEJMoa1816714

[3] Squib BM . Bristol Myers Squibb and Exelixis Announce Positive Topline Results from Pivotal Phase 3 CheckMate ‐9ER Trial Evaluating Opdivo® (nivolumab) in Combination with CABOMETYX® (cabozantinib) in Previously Untreated Advanced Renal Cell Carcinoma Princneton. NJ. 2020.

[4] Sherman RE , Anderson SA , Dal Pan GJ , et al. Real‐world evidence — what is it and what can it tell us? N Engl J Med. 2016;375:2293‐2297. 27959688 10.1056/NEJMsb1609216

[5] Eisenhauer EA , Therasse P , Bogaerts J , et al. New response evaluation criteria in solid tumours: revised RECIST guideline (version 1.1). Eur J Cancer. 2009;45:228‐247. 19097774 10.1016/j.ejca.2008.10.026

[6] Wallis CJD , Mahar AL , Satkunasivam R , et al. Cardiovascular and skeletal‐related events following localized prostate cancer treatment: role of surgery, radiotherapy, and androgen deprivation. Urol. 2016;97:145‐152. 27502032 10.1016/j.urology.2016.08.002

[7] Atkins MB , Plimack ER , Puzanov I , et al. Axitinib in combination with pembrolizumab in patients with advanced renal cell cancer: a non‐randomised, open‐label, dose‐finding, and dose‐expansion phase 1b trial. Lancet Oncol. 2018;19:405‐415. 29439857 10.1016/S1470-2045(18)30081-0 PMC6860026

[8] McDermott DF , Infante JR , Chowdhury S , et al. 2622 a phase I/II study to assess the safety and efficacy of pazopanib (paz) and pembrolizumab (pembro) in patients (pts) with advanced renal cell carcinoma (aRCC). Eur J Cancer. 2015;51:S519‐S520. 10.1016/j.clgc.2021.04.007 PMC9494291 34006498

[9] Taylor M , Dutcus CE , Schmidt E , et al. A phase 1b trial of lenvatinib (LEN) plus pembrolizumab (PEM) in patients with selected solid tumors. Ann Oncol. 2016;27.

[10] Lee C‐H , Makker V , Rasco DW , et al. Lenvatinib + pembrolizumab in patients with renal cell carcinoma: updated results. J Clin Oncol. 2018;36:4560.

[11] Ko JJ , Xie W , Kroeger N , et al. The international metastatic renal cell carcinoma database consortium model as a prognostic tool in patients with metastatic renal cell carcinoma previously treated with first‐line targeted therapy: a population‐based study. Lancet Oncol. 2015;16:293‐300. 25681967 10.1016/S1470-2045(14)71222-7

[12] Hughes PE , Caenepeel S , Wu LC . Targeted therapy and checkpoint immunotherapy combinations for the treatment of cancer. Trends Immunol. 2016;37:462‐476. 27216414 10.1016/j.it.2016.04.010

[13] Alfaro C , Suarez N , Gonzalez A , et al. Influence of bevacizumab, sunitinib and sorafenib as single agents or in combination on the inhibitory effects of VEGF on human dendritic cell differentiation from monocytes. Br J Cancer. 2009;100:1111. 19277038 10.1038/sj.bjc.6604965 PMC2670006

[14] Gabrilovich DI , Chen HL , Girgis KR , et al. Production of vascular endothelial growth factor by human tumors inhibits the functional maturation of dendritic cells. Nat Med. 1996;2:1096‐1103. 8837607 10.1038/nm1096-1096

[15] Gabrilovich DI , Ishida T , Nadaf S , Ohm JE , Carbone DP . Antibodies to vascular endothelial growth factor enhance the efficacy of cancer immunotherapy by improving endogenous dendritic cell function. Clin Cancer Res. 1999;5:2963‐2970. 10537366

[16] Dirkx AEM , Egbrink MGAO , Castermans K , et al. Anti‐angiogenesis therapy can overcome endothelial cell anergy and promote leukocyte‐endothelium interactions and infiltration in tumors. FASEB J. 2006;20:621‐630. 16581970 10.1096/fj.05-4493com

[17] Hamzah J , Jugold M , Kiessling F , et al. Vascular normalization in Rgs5‐deficient tumours promotes immune destruction. Nature. 2008;453:410. 18418378 10.1038/nature06868

[18] Yasuda S , Sho M , Yamato I , et al. Simultaneous blockade of programmed death 1 and vascular endothelial growth factor receptor 2 (VEGFR2) induces synergistic anti‐tumour effect in vivo. Clin Exp Immunol. 2013;172:500‐506. 23600839 10.1111/cei.12069 PMC3646450

[19] Guislain A , Gadiot J , Kaiser A , et al. Sunitinib pretreatment improves tumor‐infiltrating lymphocyte expansion by reduction in intratumoral content of myeloid‐derived suppressor cells in human renal cell carcinoma. Cancer Immunol Immunother. 2015;64:1241‐1250. 26105626 10.1007/s00262-015-1735-z PMC11028512

[20] Liu X‐D , Hoang A , Zhou L , et al. Resistance to antiangiogenic therapy is associated with an immunosuppressive tumor microenvironment in metastatic renal cell carcinoma. Cancer Immunol Res. 2015;3:1017‐1029. 26014097 10.1158/2326-6066.CIR-14-0244 PMC4561186

[21] Choueiri TK , Apolo AB , Powles T , et al. A phase 3, randomized, open‐label study of nivolumab combined with cabozantinib vs sunitinib in patients with previously untreated advanced or metastatic renal cell carcinoma (RCC; CheckMate 9ER). J Clin Oncol. 2018;36:TPS4598

[22] Nadal RM , Mortazavi A , Stein M , et al. Results of phase I plus expansion cohorts of cabozantinib (Cabo) plus nivolumab (Nivo) and CaboNivo plus ipilimumab (Ipi) in patients (pts) with with metastatic urothelial carcinoma (mUC) and other genitourinary (GU) malignancies. J Clin Oncol. 2018;36:515.29267131

[23] Choueiri TK , Escudier B , Powles T , et al. Cabozantinib versus everolimus in advanced renal‐cell carcinoma. N Engl J Med. 2015;373:1814‐1823. 26406150 10.1056/NEJMoa1510016 PMC5024539

[24] Motzer RJ , Escudier B , McDermott DF , et al. Nivolumab versus everolimus in advanced renal‐cell carcinoma. N Engl J Med. 2015;373:1803‐1813. 26406148 10.1056/NEJMoa1510665 PMC5719487

[25] Shah AY , Kotecha RR , Lemke EA , et al. Outcomes of patients with metastatic clear‐cell renal cell carcinoma treated with second‐line VEGFR‐TKI after first‐line immune checkpoint inhibitors. Eur J Cancer. 2019;114:67‐75. 31075726 10.1016/j.ejca.2019.04.003 PMC7537491

[26] Amin A , Plimack ER , Infante JR , et al. Nivolumab (anti‐PD‐1; BMS‐936558, ONO‐4538) in combination with sunitinib or pazopanib in patients (pts) with metastatic renal cell carcinoma (mRCC). J Clin Oncol. 2014;32:5010.

[27] Lewis JH . The adaptive response (drug tolerance) helps to prevent drug‐induced liver injury. Gastroenterolo Hepatol. 2012;8:333‐336. PMC3424431 22933867

[28] Yu‐Li SUH . Reintroducing pazopanib reverses the primary resistance of nivolumab in a patient with metastatic clear‐cell renal cell carcinoma. Clin Genitourin Cancer. 2018;16:114‐116. 29329663 10.1016/j.clgc.2017.12.002

[29] Taylor MH , Lee C‐H , Makker V , et al. Phase IB/II trial of lenvatinib plus pembrolizumab in patients with advanced renal cell carcinoma, endometrial cancer, and other selected advanced solid tumors. J Clin Oncol. 2020;38:1154‐1163. 31961766 10.1200/JCO.19.01598 PMC7145588

